# Supplementary material for: The interactome of intact mitochondria by cross-linking mass spectrometry provides evidence for coexisting respiratory supercomplexes
Source: Mol Cell Proteomics. 2017 Dec 8;17(2):216–32. doi: 10.1074/mcp.RA117.000470 (PMC5795388; doi:10.1074/mcp.RA117.000470)
Supplement: Supplemental Data [file supp_RA117.000470_133922_0_supp_23978_fzffwf.zip › spectra_annotation/mito_spectra_annotation/353-1-1-1-11-1.pdf]

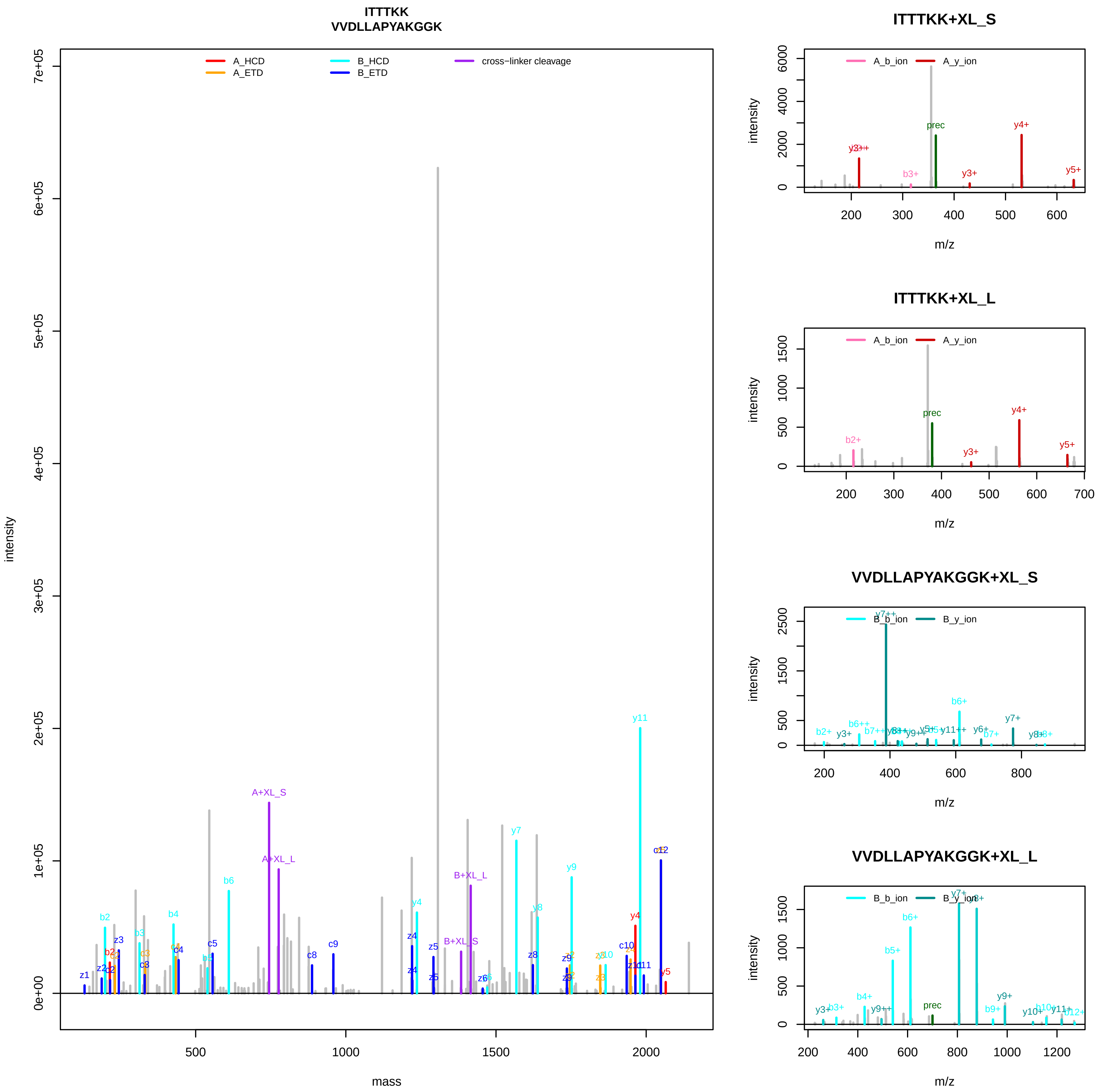

ITTTKK
VVDLLAPYAKGGK
A_HCD
A_ETD
B_HCD
B_ETD
cross−linker cleavage
intensity
mass
A+XL_S
A+XL_L
B+XL_L
B+XL_S
y11
c12
y9
y7
y8
y4
y5
b6
b4
b3
b2
c10
c11
c9
c8
c5
c4
c3
z1
z2
z3
z4
z5
z6
z8
z9
ITTTKK+XL_S
ITTTKK+XL_L
VVDLLAPYAKGGK+XL_S
VVDLLAPYAKGGK+XL_L
A_b_ion
A_y_ion
B_b_ion
B_y_ion
prec
y3++
y4+
y3+
y5+
b3+
b2+
y7++
b6+
y7+
b6++
b5+
b4+
y9+
m/z
